# The association between *Interleukin-6* rs1800795/rs1800797 polymorphisms and risk of rotator cuff tear in a Chinese population

**DOI:** 10.1042/BSR20200193

**Published:** 2020-04-21

**Authors:** Jin Li, Lifeng Jiang, Xindie Zhou, Lidong Wu, Dong Li, Gang Chen

**Affiliations:** 1 Department of Orthopedic Surgery, The Second Affiliated Hospital of Jiaxing University, Jiaxing 314000, China; 2 Department of Orthopedic Surgery, The Second Affiliated Hospital, Zhejiang University School of Medicine, Hangzhou 310000, China; 3 Department of Orthopedics, The Affiliated Changzhou No.2 People’s Hospital of Nanjing Medical University, Changzhou 213000, China; 4 Department of Trauma Center, The Affiliated Changzhou No.2 People’s Hospital of Nanjing Medical University, Changzhou 213000, China

**Keywords:** follow-up, Interleukin-6, polymorphism, rotator cuff tear

## Abstract

Expression of proinflammatory cytokines, such as interleukin (IL)-6 (IL-6) and metalloproteases, are elevated in patients with rotator cuff tear (RCT). In order to investigate the role of *IL-6* gene polymorphisms on RCT risk, we genotyped two SNPs on *IL-6* gene (rs1800795 and rs1800797) in 138 RCT patients and 137 healthy controls using polymerase chain reaction (PCR) and Sanger sequencing. The IL-6 expression in shoulder joint synovial fluid was determined by using enzyme-linked immunosorbent assay (ELISA) method. The constant score and visual analog scale (VAS) were used to evaluate the clinical outcome of two s (surgicsal vs. conservative) for RCT patients. For rs1800795, individuals with the GG genotype or G allele had significantly higher risk of RCT. Elevated risk of tear size was associated with the GG genotype of the rs1800795 polymorphism. The IL-6 rs1800797 polymorphism was also associated with an increased risk of RCT, especially among female, drinkers, and individuals with B(MI) < 25 kg/m^2^. The elevated levels of *IL-6* gene were observed among the mutant genotype of rs1800795/rs1800797 polymorphism. Surgical group is significantly better than conservative treatment from the perspective of constant score and VAS. Furthermore, CG genotype of rs1800795 polymorphism increased the constant score at 6 months in comparison with CC genotype. In conclusion, our study supports a role of IL-6 rs1800795/rs1800797 polymorphisms on increased RCT risk. The RCT patients with CG genotype of rs1800795 polymorphism have more obvious surgical treatment effects by influencing the IL-6 expression.

## Introduction

Rotator cuff disease is the most common etiology of shoulder pain, which leads to discomfort, pain and motor dysfunction [1]. Rotator cuff tear (RCT) is present in 20–54% of persons aged between 60 and 80 years [2,3]. As the population ages, the prevalence of RCTs increases significantly [4]. There is evidence that metabolic disorders (diabetes, obesity, hypercholesterolemia), hypertension and cigarette smoking are risk factors for RCT development [5]. In addition to behavioral influences, familial and genetic factors that influence RCT development have also been identified [6].

Patients with full-thickness RCT have increased levels of synovial inflammation in comparison with healthy controls [7]. Interleukin (IL)-6 (IL-6), which is rapidly and transiently produced in response to infections and tissue injuries, contributes to host defense [8]. In patients with RCT, expression of IL-1, IL-6 and TNF-α in the subacromial bursa has been shown to be elevated in comparison with healthy controls [9]. Additionally, higher IL-1β and IL-6 levels were related to more severe pain in animal RCT models [10]. IL-6 expression levels are higher in ruptured Achilles tendon compared with normal Achilles tendon and painful Achilles tendon [11].

IL-6 is located on chromosome 7p15.3 and has six exons. *IL-6* gene variants have been studied in various diseases. However, little is known about their association with susceptibility to RCT. The rs1800795 (−174 G>C) and rs1800797 (−597 A>G) polymorphisms are located in the promoter region of *IL-6* gene, and these polymorphisms influence the production of IL-6 in plasma [12,13]. As such, we performed a case–control study to evaluate the role of *IL-6* gene polymorphisms (rs1800795 and rs1800797) on the risk of RCT in a Chinese population. Additionally, the study also evaluated the association between IL-6 gene polymorphisms and clinical outcome of two management of RCT (operative vs. consevative).

## Methods

### Study subjects

One hundred and thirty-eight patients were recruited for the present study and were screened with magnetic resonance imaging to confirm full-thickness supraspinatus or infraspinatus RCT at the Second Affiliated Hospital of Jiaxing University and The Second Affiliated Hospital, Zhejiang University School of Medicine between October 2015 and December 2018. Exclusion criteria included partial-thickness RCT, calcifying tendinitis, trauma or systemic disease, or any patient who had undergone a surgical procedure on the injured shoulder. The control group (matched for age and sex) consisted of individuals receiving a routine health examination at the same hospitals and without symptoms of shoulder pain and clinical signs of rotator cuff diseases. Participants with prior operations on their shoulder or a history of humeral fracture were excluded from the present study. All participants completed self-designed questionnaire forms (sex, age, drinking status, smoking status and body mass index (BMI)) that included medication use, personal history of rotator cuff disease and inflammatory disease.

The present study was approved by the Ethics Committee of the above two hospitals. All participants provided written informed consent prior to participation in the study.

### DNA extraction and genotyping

We extracted genomic DNA from 2 ml whole blood sample using the QIAamp DNA Blood Mini Kit (Qiagen, Hilden, Germany) according to the manufacturer’s instructions. Optical density at 260/280 nm was measured to evaluate the quality of the extracted DNA. Genotyping was performed by polymerase chain reaction (PCR) and Sanger sequencing. The primers were: 5′-CTGGCAGCACAAGGCAAAC-3′ (rs1800797F) and 5′-AGGCAACTGGACCGAAGG-3′ (rs1800795R). PCR amplification was performed in a 25-µl reaction volume containing 200 μM dNTPs, 10× PCR buffer of 1.5 μM MgCl_2_, 1 U Taq polymerase, 100 ng genomic DNA and 20 pmol of each pair of primer and ddH_2_O. The PCR product was analyzed on 2% agarose gel electrophoresis with ethidium bromide and was then photographed. The purified PCR product was sequenced by GenScript Inc. (Nanjing, China). Genotyping on a random selection (3%) of samples was repeated in a blind manner for quality control, and the results were 100% concordant.

### Enzyme-linked immunosorbent assay

Shoulder joint synovial fluid was collected from RCT patients. The collected synovial fluid was centrifuged and the supernatants were stored at −80°C until use. The IL-6 levels were determined using the human IL-6 enzyme-linked immunosorbent assay (ELISA) Kit (Sino Biological, Beijing, China) following manufacturer’s recommendations. The IL-6 level was calculated by interpolation from a standard curve.

### Outcome assessment

We divided RCT patients into two groups: surgical and conservative groups. The patients from surgical group was diagnosed with RCT through magnetic resonance imaging. Conservative management includes nonsteroidal anti-inflammatory drugs, physiotherapy and rest. Patients’ injured shoulder had not previously undergone surgery. We evaluate the clinical outcome at baseline, 6 and 12 months after surgical group and at inclusion for the conservative group. The clinical outcome was evaluated by constant score and visual analog scale (VAS). The constant score includes shoulder function test (65 points) and patients’ subjective assessment (35 points). The pain and disability were determined with VAS. The score varies from 0 to 9, and higher scores indicate higher levels of pain and disability.

### Statistical analyses

A Student’s *t* test or χ^2^ test was used to assess differences in mean and frequency distributions of demographic and clinical characteristics among cases and controls. We calculated the odds ratios (ORs) and 95% confidence intervals (CIs) to evaluate the association between IL-6 gene polymorphisms and risk of RCT by logistic regression analyses, and adjusted for sex and age. A goodness-of-fit chi-squared test was used to test the departure from Hardy–Weinberg equilibrium (HWE) of IL-6 genotype distributions in controls. All statistical analyses were performed using SAS (var. 9.1.3; SAS Institute, Cary, NC, U.S.A.). *P*<0.05 was considered to indicate statistical significance.

## Results

### Characteristics of the study population

One hundred and thirty-eight RCT patients and 137 healthy controls were recruited in the present study. The average age of the RCT patients was 52.66 years compared with 53.12 years for the controls (*P*=0.128). There was no significant difference between the two groups with regard to sex, smoking status, alcohol consumption, BMI. We also recorded family history of RCT and tear size of RCT patients to investigate its association with IL-6 rs1800795/rs1800797 polymorphisms (Table 1).

**Table 1:** The demographics and risk factors of study population

Characteristics	Case (*n*=138)	Control (*n*=137)	*P*
Age, years	52.66 ± 10.98	53.12 ± 11.05	0.731
Sex			0.184
Male	75 (54.3%)	64 (46.7%)	
Female	63 (45.7%)	73 (53.3%)	
BMI, kg/m^2^	25.87 ± 3.18	25.99 ± 3.21	0.757
Smoking			0.763
Yes	68 (49.3%)	70 (51.1%)	
No	70 (50.7%)	67 (48.9%)	
Alcohol			0.764
Yes	66 (47.8%)	68 (49.6%)	
No	72 (51.2%)	69 (50.4%)	
Affected side			
Right	80 (58.0%)		
Left	58 (42.0%)		
Family history of RCT			
Yes	11 (8.0%)		
No	127 (92.0%)		
Tear size			
Small	71 (51.4%)		
Medium	41 (29.7%)		
Large	26 (18.8%)		
Pre-operative stiffness			
Yes	38 (27.5%)		
No	100 (72.5%)		

### Quantitative analysis

The genotype distributions of *IL-6* gene polymorphisms among cases and controls are shown in Table 2. No significant deviation from HWE was found for rs1800795/rs1800797 polymorphisms in both cases and controls, suggesting these subjects are representative of the local population. The *IL-6* rs1800795 polymorphism was significantly associated with an increased risk of RCT under the homozygous and allelic models. Moreover, the GG or GG+AG genotype of *IL-6* rs1800797 polymorphism showed significant correlation with risk of RCT compared with AA genotype (GG vs. AA; OR, 2.29; 95% CI, 1.11–4.72; *P*=0.025; GG+AG vs. AA; OR, 1.69; 95% CI, 1.03–2.79; *P*=0.040). The *IL-6* rs1800797 polymorphism demonstrated significant association with risk of RCT under the allelic model. After adjusting for sex age and BMI, these findings remained significant.

**Table 2:** Genotype frequencies of IL-6 gene polymorphisms in cases and controls

Models	Genotype	Case (*n*, %)	Control (*n*, %)	OR (95% CI)	*P*-value	*OR (95% CI)	*P-value
rs1800795							
Wild	CC	39 (28.5%)	54 (39.7%)	1.00 (reference)	*-*	1.00 (reference)	-
Heterozygote	CG	69 (50.4%)	64 (47.1%)	1.49 (0.88–2.55)	0.141	1.48 (0.86–2.54)	0.155
Homozygote	GG	29 (21.2%)	18 (13.2%)	**2.23 (1.09–4.57)**	**0.029**	**2.10 (1.01–4.33)**	**0.046**
Dominant	CC	39 (28.5%)	54 (39.7%)	1.00 (reference)	-	1.00 (reference)	-
	GG+CG	98 (71.5%)	82 (60.3%)	1.66 (0.99–2.74)	0.051	1.62 (0.97–2.70)	0.066
Recessive	CG+CC	108 (78.8%)	118 (86.8%)	1.00 (reference)		1.00 (reference)	-
	GG	29 (21.2%)	18 (13.2%)	1.76 (0.93–3.35)	0.085	1.66 (0.87–3.19)	0.126
Allele	C	147 (53.6%)	172 (63.2%)	1.00 (reference)	-	1.00 (reference)	-
	G	127 (46.4%)	100 (36.8%)	**1.49 (1.06–2.09)**	**0.023**	-	*-*
rs1800797							
Wild	AA	41 (29.9%)	57 (41.9%)	1.00 (reference)	-	1.00 (reference)	-
Heterozygote	AG	68 (49.6%)	62 (45.6%)	1.53 (0.90–2.59)	0.118	1.56 (0.91–2.66)	0.104
Homozygote	GG	28 (20.4%)	17 (12.5%)	**2.29 (1.11–4.72)**	**0.025**	**2.29 (1.10–4.77)**	**0.026**
Dominant	AA	41 (29.9%)	57 (41.9%)	1.00 (reference)	-	1.00 (reference)	-
	GG+AG	96 (70.1%)	79 (58.1%)	**1.69 (1.03–2.79)**	**0.040**	**1.72 (1.04–2.85)**	**0.036**
Recessive	AG+AA	109 (79.6%)	119 (87.5%)	1.00 (reference)	-	1.00 (reference)	-
	GG	28 (20.4%)	17 (12.5%)	1.80 (0.93–3.47)	0.080	1.78 (0.92–3.47)	0.089
Allele	A	150 (54.7%)	176 (64.7%)	1.00 (reference)	-	1.00 (reference)	-
	G	124 (45.3%)	96 (35.3%)	**1.52 (1.07–2.14)**	**0.018**	-	**-**

The genotyping was successful in 137 cases and 136 controls for rs1800795; the genotyping was successful in 137 cases and 136 controls for rs1800797. Bold values are statistically significant (*P*<0.05).

* Adjusted for age, sex and BMI.

We performed subgroup analyses according to sex, smoking status, alcohol consumption, ageand BMI (Table 3). For rs1800795, there were no significant differences in the frequencies of genotypes and alleles for the rs1800795 polymorphism between these groups. However, we found a significant positive association between *IL-6* rs1800797 polymorphism and RCT risk among women and drinkers in the homozygous model. This significant association was also evident in subgroups of subjects with BMI < 25 kg/m^2^.

**Table 3:** Stratified analyses between rs1800795/rs1800797 polymorphisms and the risk of RCT

Variable	Case/control			Heterozygous	Homozygous	Recessive	Dominant
	CC	CG	GG	CG vs. CC OR (95% CI), *P*-value	GG vs. CC OR (95% CI), *P*-value	GG vs. CC+CG OR (95% CI), *P*-value	GG+CG vs. CC OR (95% CI), *P*-value
rs1800795							
Sex							
Male	20/26	36/27	18/10	1.73 (0.81–3.73); 0.160	2.34 (0.89–6.16); 0.085	1.70 (0.72–4.02); 0.224	1.90 (0.93–3.89); 0.080
Female	19/28	33/37	11/8	1.31 (0.62–2.78); 0.474	2.03 (0.69–5.97); 0.200	1.72 (0.64–4.58); 0.279	1.44 (0.70–2.95); 0.317
Smoking							
Yes	22/30	36/33	9/7	1.49 (0.72–3.07); 0.283	1.76 (0.57–5.43); 0.330	1.40 (0.49–3.99); 0.533	1.53 (0.77–3.08); 0.228
No	17/24	33/31	20/11	1.50 (0.68–3.32); 0.313	2.57 (0.98–6.72); 0.055	2.00 (0.87–4.58); 0.102	1.78 (0.85–3.74); 0.127
Alcohol							
Yes	20/28	35/34	10/6	1.44 (0.69–3.03); 0.335	2.33 (0.73–7.47); 0.154	1.88 (0.64–5.51); 0.250	1.58 (0.77–3.22); 0.213
No	19/26	34/30	19/12	1.55 (0.72–3.35); 0.263	2.17 (0.85–5.51); 0.105	1.67 (0.74–3.78); 0.216	1.73 (0.84–3.54); 0.135
Age (years)							
<60	29/37	51/42	19/13	1.55 (0.82–2.92); 0.177	1.87 (0.79–4.39); 0.154	1.44 (0.67–3.12); 0.351	1.62 (0.89–2.96); 0.114
≥60	10/17	18/22	10/5	1.39 (0.51–3.78); 0.517	3.40 (0.90–12.82); 0.071	2.79 (0.86–9.05); 0.088	1.76 (0.69–4.53); 0.239
BMI (kg/m^2^)							
<25	15/21	33/20	13/9	2.20 (0.92, 5.25); 0.076	1.93 (0.65, 5.68); 0.235	1.20 (0.47, 3.11); 0.701	2.12 (0.94, 4.78); 0.072
≥25	24/33	36/44	16/25	1.13 (0.57, 2.23); 0.736	2.44 (0.93, 6.46); 0.071	2.28 (0.94, 5.52); 0.067	1.35 (0.70, 2.59); 0.367

Variable	case/control			Heterozygous	Homozygous	Recessive	Dominant
	AA	AG	GG	AG vs. AA OR (95% CI), *P*-value	GG vs. AA OR (95% CI), *P*-value	GG vs. AA+AG OR (95% CI), *P*-value	GG+AG vs. AA OR (95% CI), *P*-value
rs1800797							
Sex							
Male	25/25	34/30	15/9	1.13 (0.54–2.38); 0.740	1.67 (0.62–4.51); 0.314	1.55 (0.63–3.84); 0.340	1.26 (0.63–2.52); 0.520
Female	16/32	34/32	13/8	2.13 (0.98–4.59); 0.055	3.25 (1.12–9.43); 0.030	2.08 (0.80–5.41); 0.133	2.35 (1.13–4.89); 0.022
Smoking							
Yes	18/25	36/36	14/9	1.39 (0.65–2.98); 0.398	2.16 (0.77–6.07); 0.144	1.76 (0.71–4.38); 0.227	1.54 (0.75–3.19); 0.243
No	23/32	32/26	14/8	1.71 (0.81–3.61); 0.157	2.44 (0.88–6.76); 0.088	1.85 (0.72–4.74); 0.203	1.88 (0.94–3.77); 0.075
Alcohol							
Yes	15/26	32/33	15/9	1.89 (0.86–4.17); 0.115	2.89 (1.02–8.19); 0.046	1.93 (0.78–4.78); 0.156	2.11 (0.99–4.48); 0.054
No	26/31	36/29	13/8	1.32 (0.64–2.71); 0.458	1.94 (0.70–5.39); 0.205	1.68 (0.65–4.35); 0.285	1.45 (0.74–2.86); 0.283
Age (years)							
<60	32/37	49/44	18/10	1.29 (0.69–2.40); 0.427	2.08 (0.84–5.15); 0.113	1.80 (0.78–4.14); 0.166	1.44 (0.79–2.60); 0.233
≥60	9/20	19/18	10/7	2.35 (0.85–6.49); 0.101	3.17 (0.91–11.03); 0.069	1.94 (0.66–5.72); 0.231	2.58 (0.99–6.67); 0.051
BMI (kg/m^2^)							
<25	15/23	32/20	15/6	2.58 (1.09, 6.12); 0.031	3.83 (1.22, 12.09); 0.021	2.23 (0.79, 6.28); 0.128	2.88 (1.28, 6.49); 0.011
≥25	26/34	36/42	13/11	1.12 (0.57, 2.21); 0.741	1.55 (0.60, 4.00); 0.370	1.45 (0.61, 3.46); 0.404	1.21 (0.64, 2.30); 0.562

Bold values are statistically significant (*P*<0.05).

Most clinical characteristics of RCT patients did not demonstrate and significant association with the *IL-6* rs1800795 polymorphism (Table 4). However, the GG genotype of rs1800795 polymorphism was more frequent in patients with large tear size compared with patients with medium tear size, suggesting an association with tear size of RCT patients. Additionally, we observed no evidence of a significant association between rs1800797 polymorphism and clinical characteristics of RCT.

**Table 4:** The associations between IL-6 rs1800795/rs1800797 polymorphisms and clinical characteristics of RCT

Characteristics	Genotype distributions			
	CC	CG	GG	CG+GG
rs1800795				
Tear size				
Large/Medium	4/15	12/21	10/5	14/26
OR (95% CI); *P*-value	1.0 (reference)	2.14 (0.58–7.95); 0.255	**7.50 (1.61–34.95); 0.010**	2.02 (0.56–7.26); 0.282
Tear size				
Large/Small	4/20	12/36	10/14	22/50
OR (95% CI); *P*-value	1.0 (reference)	1.67 (0.47–5.86); 0.426	3.57 (0.93–13.72); 0.064	2.20 (0.67–7.19); 0.192
Family history				
Yes/No	1/38	8/61	2/27	10/88
OR (95% CI); *P*-value	1.0 (reference)	4.98 (0.60–41.43); 0.137	2.82 (0.24–32.64); 0.408	4.32 (0.53–34.93); 0.170

Characteristics	Genotype distributions			
	AA	AG	GG	AG+GG
rs1800797				
Tear size				
Large/Medium	4/12	16/18	5/11	21/29
OR (95% CI); *P*-value	1.0 (reference)	2.67 (0.72–9.95); 0.144	1.36 (0.29–6.42); 0.695	2.17 (0.61–7.68); 0.229
Tear size				
Large/Small	4/25	16/34	5/12	21/46
OR (95% CI); *P*-value	1.0 (reference)	2.94 (0.88–9.88); 0.081	2.60 (0.59–11.49); 0.206	2.85 (0.88–9.24); 0.080
Family history				
Yes/No	1/40	6/62	4/24	10/86
OR (95% CI); *P*-value	1.0 (reference)	3.87 (0.45–33.37); 0.218	6.67 (0.70–63.19); 0.098	4.65 (0.58–37.59); 0.149

Bold values are statistically significant (*P*<0.05).

### IL-6 expression in shoulder joint synovial fluid

**Figure 1:**
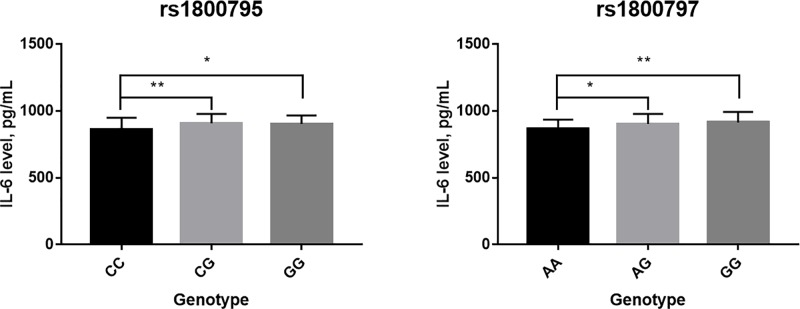
The IL-6 expression among three genotypes (**A**) rs1800795; (**B**) rs1800797.*P<0.05; **P<0.01.

We further investigated the effect of IL-6 gene polymorphism on the IL-6 expression using ELISA method. There is a significant difference among three genotypes of rs1800795/rs1800797 polymorphism with regard to the IL-6 levels (Figure 1). The mutant/heterozygous genotype had a higher IL-6 levels had a significantly higher expression of IL-6 in shoulder joint synovial fluid than wild genotype.

### Outcome

A majority of RCT patients chose surgical treatment rather than conservative treatment (Table 5). No significant difference was observed among two groups with regard to age, sex, drinking, affected side, family history of RCT and tear size. Remarkably, the mean BMI and proportion of smokers differ significantly among two groups.

**Table 5:** The functional baseline characteristics of patients with RCT

Variables	Surgery group (*n*=89)	Conservative group (*n*=49)	*P*
Age, years	53.34 ± 10.69	51.43 ± 11.50	0.330
Sex			0.895
Male	48 (53.9%)	27 (55.1)	
Female	41 (46.1%)	22 (44.9)	
BMI, kg/m^2^	25.45 ± 3.13	26.64 ± 3.16	**0.037**
Smoking			**0.037**
Yes	38 (42.7%)	30 (61.2%)	
No	51 (57.3%)	19 (38.8%)	
Alcohol			0.104
Yes	38 (42.7%)	28 (57.1%)	
No	51 (57.3%)	21 (42.9%)	
Affected side			0.830
Right	51 (57.3%)	29 (59.2%)	
Left	38 (42.7%)	20 (40.8%)	
Family history of RCT			0.211
Yes	9 (10.1%)	2 (4.1%)	
No	80 (89.9%)	47 (95.9%)	
Tear size			0.263
Small	42 (47.2%)	29 (59.2%)	
Medium	27 (30.3%)	14 (28.6%)	
Large	20 (22.5%)	6 (12.2%)	
Constant score (range 0–100)			
Baseline	36.94 ± 3.44	37.93 ± 3.40	0.107
6 months	66.05 ± 3.79	64.71 ± 3.28	**0.042**
12 months	81.90 ± 3.77	77.81 ± 2.49	**<0.001**
VAS score (range 0–10)			
Baseline	6.74 ± 0.44	6.56 ± 0.38	**0.017**
6 months	2.62 ± 0.31	3.30 ± 0.38	**<0.001**
12 months	1.09 ± 0.37	2.76 ± 0.44	**<0.001**

Bold values are statistically significant (*P*<0.05).

Table 5 also presents the follow-up results of RCT patients with/without surgery treatment. There was no significant difference in baseline constant score between surgery and conservative groups. The constant score of the surgery group was significantly higher than that of the conservation group (*P*<0.05) after 6 months or 1 year of treatment. The primary VAS in the surgery group was higher, but decreased significantly after surgery treatment. Above all, the surgical treatment was significantly better than conservation treatment through evaluating constant score and VAS.

The present study also assessed whether IL-6 gene polymorphism is associated with the clinical outcome of RCT patients receiving surgery treatment (Table 6). The basic constant score in three genotypes of rs1800795 polymorphism did not differ significantly. Notably, CG genotype of rs1800795 polymorphism has the highest constant score compared with CC or GG genotype at 6 months (*P*<0.05), indicating the significant association between IL-6 rs1800795 polymorphism. However, the finding is not applicable to rs1800797 polymorphism.

**Table 6:** The association between IL-6 gene polymorphisms and functional outcome of surgery group at 1 year follow-up

Outcome	Genotype			*P*
	Wild-type	Heterozygote	Homozygous	
	CC (*n*=21)	CG (*n*=47)	GG (*n*=20)	
**rs1800795**				
Constant score (range 0–100)				
Baseline	37.03 ± 3.97	36.69 ± 3.23	37.36 ± 3.54	0.764
6 months	64.35 ± 3.03	67.02 ± 3.82	65.82 ± 3.80	**0.023**
12 months	81.06 ± 3.58	82.22 ± 3.89	82.27 ± 3.64	0.463
VAS score (range 0–10)				
Baseline	6.77 ± 0.50	6.66 ± 0.40	6.88 ± 0.43	0.170
6 months	2.55 ± 0.32	2.64 ± 0.32	2.63 ± 0.28	0.570
12 months	1.13 ± 0.38	1.13 ± 0.37	0.99 ± 0.36	0.296

Outcome	Genotype			*P*
	Wild-type	Heterozygote	Homozygous	
	AA (*n*=28)	AG (*n*=45)	GG (*n*=15)	
**rs1800797**				
Constant score (range 0–100)				
Baseline	37.50 ± 3.98	36.66 ± 3.06	36.56 ± 3.63	0.553
6 months	66.05 ± 3.79	66.18 ± 4.11	65.70 ± 3.05	0.916
12 months	82.56 ± 3.44	81.62 ± 3.81	81.89 ± 4.18	0.576
VAS score (range 0–10)				
Baseline	6.69 ± 0.50	6.78 ± 0.42	6.70 ± 0.38	0.625
6 months	2.65 ± 0.26	2.61 ± 0.33	2.59 ± 0.35	0.770
12 months	0.99 ± 0.38	1.15 ± 0.32	1.15 ± 0.47	0.175

## Discussion

Findings from the present study suggest that the *IL-6* rs1800795/rs1800797 polymorphisms are associated with an increased risk of RCT. The subgroup analyses performed suggest that the increased effect of rs1800797 polymorphism on RCT is stronger in women, drinkers and subjects with BMI < 25 kg/m^2^. The *IL-6* rs1800795 polymorphism was correlated with tear size of RCT patients. Additionally, the RCT patients with CG genotype of rs1800795 polymorphism have more obvious surgical treatment effects.

RCT rate increases after the age of 60 [14]. With advancing age, the cuff undergoes several internal changes, such as collagen disorganization and thinning, myxoid and hyaline degeneration, fatty infiltration and vascular proliferation [15]. In addition, the type II collagen variant at the fibrocartilage junctions of the tendon, which is primarily responsible for compressive load, converts into type III [16]. This decreases the capability of the tendon to withstand compressive loads and predisposes it to tear [16]. Moreover, IL-6 and/or soluble IL-6 receptor can down-regulate human type Ⅱ collagen gene expression in articular chondrocytes [17].

The association between *IL-6* rs1800795 polymorphism and disease risk such as cervical cancer [18]; polycystic ovary syndrome [19]; type 2 diabetes [20] and rheumatoid arthritis [21], has been widely investigated. Similarly, *IL-6* rs1800797 polymorphism has also been studied in various disease [22–25]. However, few studies have investigated its possible association with RCT susceptibility. Patients with full-thickness RCT have greater levels of synovial inflammation compared with controls and IL-6 is significantly increased in individuals with RCT [7]. In the present study, we conducted a hospital-based case–control study to evaluate the role of *IL-6* gene polymorphisms on the risk of RCT. *IL-6* rs1800795 polymorphism conferred susceptibility to RCT and was associated with the tear size. Additionally, we found a significant association between rs1800797 polymorphism and RCT risk, which appeared stronger in women, drinkers and subjects with BMI < 25 kg/m^2^. To date, the present study represents the first to examine the association between *IL-6* gene variants and RCT risk and may help guide future studies in this area.

Piper et al. [26] conducted a meta-analysis with three Caucasian studies to evaluate operative versus nonoperative treatment for the management of full-thickness RCT. They observed statistically significant differences favoring surgery in both constant and VAS score [26]. The clinical outcome of surgery is significantly better than those of conservative treatment [26], which is similar to our results. Notably, we also evaluate the association between IL-6 gene polymorphisms and constant score and VAS for RCT patients. We found that the constant score of CG genotype of rs1800795 polymorphism significantly improved after 6 months of surgical treatment. Further studies with larger sample size was needed to verify the finding.

There are several limitations to the present study. First, limited availability to the original dataset prevented detection for gene–environment interactions, such as diet and lifestyle. Second, the sample size of the present study was relatively small, as so we cannot rule out the possibility of false-positive or false-negative results. Later, the multicenter studies with larger sample size were need to confirm these findings. Third, two polymorphisms cannot represent the whole *IL-6* gene and may have limited impact on the development of RCT. Lastly, his was a hospital-based case–control study, selection bias was unavoidable and the participants may not represent the whole population.

In conclusion, the *IL-6* rs1800795 or rs1800797 polymorphism showed a statistically significant association with RCT risk, and these polymorphisms may serve as potential biomarkers for early screening and treatment of RCT. Future investigations that include a larger sample size, as well as functional evaluation of the studied polymorphism, are warranted to confirm the findings of the present study.

## Abbreviations

BMI: body mass index
CI: confidence interval
ELISA: enzyme-linked immunosorbent assay
HWE: Hardy–Weinberg equilibrium
IL: interleukin
OR: odds ratio
PCR: polymerase chain reaction
RCT: rotator cuff tear
VAS: visual analog scale

## References

[1] Mitchell C., Adebajo A., Hay E. and Carr A. (2005) Shoulder pain: diagnosis and management in primary care. BMJ 331, 1124–1128 10.1136/bmj.331.7525.1124 16282408 PMC1283277

[2] Bartolozzi A., Andreychik D. and Ahmad S. (1994) Determinants of outcome in the treatment of rotator cuff disease. Clin. Orthop. Relat. Res. 308, 90–97 10.1097/00003086-199411000-00015 7955708

[3] Kukkonen J., Joukainen A., Lehtinen J., Mattila K.T., Tuominen E.K., Kauko T. et al. (2015) Treatment of nontraumatic rotator cuff tears: a randomized controlled trial with two years of clinical and imaging follow-up. J. Bone. Joint Surg. Am. 97, 1729–1737 10.2106/JBJS.N.01051 26537160

[4] Gumina S., Carbone S., Campagna V., Candela V., Sacchetti F.M. and Giannicola G. (2013) The impact of aging on rotator cuff tear size. Musculoskelet. Surg. 97, 69–72 10.1007/s12306-013-0263-2 23588834

[5] Abate M., Di Carlo L., Salini V. and Schiavone C. (2017) Risk factors associated to bilateral rotator cuff tears. Orthop. Traumatol. Surg. Res. 103, 841–845 10.1016/j.otsr.2017.03.027 28578100

[6] Tashjian R.Z., Granger E.K., Zhang Y., Teerlink C.C. and Cannon-Albright L.A. (2016) Identification of a genetic variant associated with rotator cuff repair healing. J. Shoulder Elbow Surg. 25, 865–872 10.1016/j.jse.2016.02.019 27066960

[7] Abrams G.D., Luria A., Carr R.A., Rhodes C., Robinson W.H. and Sokolove J. (2016) Association of synovial inflammation and inflammatory mediators with glenohumeral rotator cuff pathology. J. Shoulder Elbow Surg. 25, 989–997 10.1016/j.jse.2015.10.011 26775747

[8] Tanaka T., Narazaki M. and Kishimoto T. (2014) IL-6 in inflammation, immunity, and disease. Cold Spring Harb. Perspect. Biol. 6, a016295 10.1101/cshperspect.a016295 25190079 PMC4176007

[9] Voloshin I., Gelinas J., Maloney M.D., O'Keefe R.J., Bigliani L.U. and Blaine T.A. (2005) Proinflammatory cytokines and metalloproteases are expressed in the subacromial bursa in patients with rotator cuff disease. Arthroscopy 21, 1076.e1071–e1079 10.1016/j.arthro.2005.05.017 16171632

[10] Yamazaki H., Ochiai N., Kenmoku T., Ohtori S., Sasho T., Miyagi M. et al. (2014) Assessment of pain-related behavior and pro-inflammatory cytokine levels in the rat rotator cuff tear model. J. Orthop. Res. 32, 286–290 10.1002/jor.22486 24018624

[11] Legerlotz K., Jones E.R., Screen H.R. and Riley G.P. (2012) Increased expression of IL-6 family members in tendon pathology. Rheumatology (Oxford) 51, 1161–1165 10.1093/rheumatology/kes002 22337942 PMC3380247

[12] Bashashati M., Moradi M. and Sarosiek I. (2017) Interleukin-6 in irritable bowel syndrome: a systematic review and meta-analysis of IL-6 (-G174C) and circulating IL-6 levels. Cytokine 99, 132–138 10.1016/j.cyto.2017.08.017 28886490

[13] Rana B.K., Flatt S.W., Health D.D., Pakiz B., Quintana E.L., Natarajan L. et al. (2017) The IL-6 gene promoter SNP and plasma IL-6 in response to diet intervention. Nutrients 9, 552 10.3390/nu9060552 28555011 PMC5490531

[14] Tempelhof S., Rupp S. and Seil R. (1999) Age-related prevalence of rotator cuff tears in asymptomatic shoulders. J. Shoulder Elbow Surg. 8, 296–299 10.1016/S1058-2746(99)90148-9 10471998

[15] Hashimoto T., Nobuhara K. and Hamada T. (2003) Pathologic evidence of degeneration as a primary cause of rotator cuff tear. Clin. Orthop Relat. Res. 415, 111–120 10.1097/01.blo.0000092974.12414.22 14612637

[16] Pandey V. and Jaap Willems W. (2015) Rotator cuff tear: a detailed update. Asia Pac. J. Sports Med. Arthrosc. Rehabil. Technol. 2, 1–14 29264234 10.1016/j.asmart.2014.11.003 PMC5730646

[17] Poree B., Kypriotou M., Chadjichristos C., Beauchef G., Renard E., Legendre F. et al. (2008) Interleukin-6 (IL-6) and/or soluble IL-6 receptor down-regulation of human type II collagen gene expression in articular chondrocytes requires a decrease of Sp1.Sp3 ratio and of the binding activity of both factors to the COL2A1 promoter. J. Biol. Chem. 283, 4850–4865 10.1074/jbc.M706387200 18065760

[18] Liu H., Lyu D., Zhang Y., Sheng L. and Tang N. (2017) Association between the IL-6 rs1800795 polymorphism and the risk of cervical cancer: a meta-analysis of 1210 cases and 1525 controls. Technol. Cancer Res. Treat. 16, 662–667 10.1177/1533034616672806 27777338 PMC5665158

[19] Chen L., Zhang Z., Huang J. and Jin M. (2018) Association between rs1800795 polymorphism in the interleukin-6 gene and the risk of polycystic ovary syndrome: a meta-analysis. Medicine (Baltimore) 97, e11558 10.1097/MD.0000000000011558 30024552 PMC6086475

[20] Ghavimi R., Sharifi M., Mohaghegh M.A., Mohammadian H., Khadempar S. and Rezaei H. (2016) Lack of association between rs1800795 (-174 G/C) polymorphism in the promoter region of interleukin-6 gene and susceptibility to type 2 diabetes in Isfahan population. Adv. Biomed. Res. 5, 18 26962520 10.4103/2277-9175.175904 PMC4770613

[21] Dar S.A., Haque S., Mandal R.K., Singh T., Wahid M., Jawed A. et al. (2017) Interleukin-6-174G>C (rs1800795) polymorphism distribution and its association with rheumatoid arthritis: a case-control study and meta-analysis. Autoimmunity 50, 158–169 28010120 10.1080/08916934.2016.1261833

[22] Saxena M., Agrawal C.G., Srivastava N. and Banerjee M. (2014) Interleukin-6 (IL-6)-597 A/G (rs1800797) & -174 G/C (rs1800795) gene polymorphisms in type 2 diabetes. Indian J. Med. Res. 140, 60–68 25222779 PMC4181162

[23] Lajunen T.K., Jaakkola J.J. and Jaakkola M.S. (2016) Interleukin 6 SNP rs1800797 associates with the risk of adult-onset asthma. Genes Immun. 17, 193–198 10.1038/gene.2016.8 26938664

[24] Chou S.C., Ko H.W. and Lin Y.C. (2016) CRP/IL-6/IL-10 single-nucleotide polymorphisms correlate with the susceptibility and severity of community-acquired pneumonia. Genet. Test Mol. Biomarkers 20, 732–740 10.1089/gtmb.2016.0156 27705004

[25] George B.C., Dunnington G.L. and DaRosa D.A. (2018) Trainee autonomy and patient safety. Ann. Surg. 267, 820–822 10.1097/SLA.0000000000002599 29166357

[26] Piper C.C., Hughes A.J., Ma Y., Wang H. and Neviaser A.S. (2018) Operative versus nonoperative treatment for the management of full-thickness rotator cuff tears: a systematic review and meta-analysis. J. Shoulder Elbow Surg. 27, 572–576 10.1016/j.jse.2017.09.032 29169957

